# Properties Comparison of Ti-Al-Si Alloys Produced by Various Metallurgy Methods

**DOI:** 10.3390/ma12193084

**Published:** 2019-09-21

**Authors:** Anna Knaislová, Pavel Novák, Jaromír Kopeček, Filip Průša

**Affiliations:** 1 Department of Metals and Corrosion Engineering, University of Chemistry and Technology, Technická 5, 166 28 Prague, Czech Republic; panovak@vscht.cz (P.N.); prusaf@vscht.cz (F.P.); 2 Institute of Physics of the Czech Academy of Sciences, Na Slovance 1999/2, 182 21 Prague, Czech Republic; kopecek@fzu.cz

**Keywords:** titanium aluminides and silicides, casting, powder metallurgy

## Abstract

Melting metallurgy is still the most frequently used and simplest method for the processing of metallic materials. Some of the materials (especially intermetallics) are very difficult to prepare by this method due to the high melting points, poor fluidity, or formation of cracks and pores after casting. This article describes the processing of Ti-Al-Si alloys by arc melting, and shows the microstructure, phase composition, hardness, fracture toughness, and compression tests of these alloys. These results are compared with the same alloys prepared by powder metallurgy by the means of a combination of mechanical alloying and spark plasma sintering. Ti-Al-Si alloys processed by melting metallurgy are characterized by a very coarse structure with central porosity. The phase composition is formed by titanium aluminides and titanium silicides, which are full of cracks. Ti-Al-Si alloys processed by the powder metallurgy route have a relatively homogeneous fine-grained structure with higher hardness. However, these alloys are very brittle. On the other hand, the fracture toughness of arc-melted samples is immeasurable using Palmqvist’s method because the crack is stopped by a large area of titanium aluminide matrix.

## 1. Introduction

Titanium intermetallics with other light elements (for example aluminium or silicon) are prospective high-temperature alloys, applicable especially for construction components working at high temperatures under static loads [1,2,3,4,5]. They are considered for high-temperature service because they offer a balance of oxidation resistance and mechanical properties at higher temperatures superior to conventional titanium alloys [6,7]. Ti-Al(Si) alloys are characterized by low density, good resistance against oxidation at 600–800 °C [8,9], good thermal stability, high specific strength at high temperatures [10,11], and a favourable ratio of mechanical properties to density [12]. Alloys based on α_2_-Ti_3_Al and γ-TiAl can be used as high-temperature construction materials for aviation applications [13,14,15], but low room-temperature ductility, as well as insufficient high-temperature strength and toughness, are problems which limit their broader use [16].

The production of Ti-Al alloys (and generally intermetallic compounds) is focused on conventional casting techniques, for example, on melting in an electric arc furnace under an argon atmosphere or electroshock remelting in an inert atmosphere [17]. Despite the enormous efforts made over the last 40 years, no castings have been produced to meet the reliability and cost requirements of the aviation industry [18]. The problems are caused especially by the high reactivity of molten titanium alloys with the ceramic crucible, and hence it is needed to use cold wall crucibles. However, cold wall crucibles enable low superheating of only about 60 °C. Slow cooling rates after centrifugal casting lead to the production of castings with a coarse-grained structure and large porosity. In order to remove the internal defects, the castings are processed by the HIP (Hot Isostatic Pressing) method. This technology is used by GE for engines for Boeing, but it is very expensive [19]. 

The melting of Ti-Al alloys is a multi-step process. The process involves the melting of an alloy, casting into a mould, extended isothermal annealing of ingot and controlled rapid crystallization of the melted metal [17]. Casting is often followed by the isostatic pressing of hot castings, which has a positive effect on homogeneity, but the production is more expensive [12].

Ti-Al alloys for turbine wheels of turbochargers are now manufactured by lost-wax casting [20]. The model of the required object is made of the wax and coated by the ceramics, therefore the massive mould is made. Then the wax is melted and poured out the mould. The metallic melt is filled into the mould and the casting solidifies. The process finishes with the removal of the ceramic mould after the solidification of the alloy. Another technology is induction skull melting (ISM). This process combines the advantages of induction melting and cold crucible melting. ISM is used for melting highly reactive material (for example TiAl) with high purity. According to this method, γ-TiAl alloys are melted in the vacuum induction furnace in a water-cooled crucible. The contamination from the crucible into the alloy is minimal. The big advantage is the continuous stirring, which guarantees the composition’s uniformity [21,22]. This process is very energy-intensive and highly expensive because of the high cost of melting furnace and its non-ecological operation, high consumption of cooling water and inert gas and low achievable melt overheating [20]. The problems of cast Ti-Al alloys also include the reactions of titanium and aluminium with the atmosphere of the melting facility, the reaction of titanium with melting crucibles or ceramic moulds, or the evaporation of aluminium. The γ-TiAl alloys prepared by this process have a polycrystalline lamellar microstructure. If the sample is heated to a temperature above 1150 °C, the polycrystalline lamellar structure is replaced by a fine homogeneous duplex one with uniform grains. The lamellar structure is less ductile than the duplex one, but it has better fracture toughness, fatigue resistance, and creep strength at high temperatures [20]. However, it is also very difficult to forge the Ti-Al alloys due to inherent poor deformability even at temperatures above 1000 °C [23,24]. Chesnutt et al. wrote that it is possible to form α_2_-TiAl and γ-TiAl alloys (with difficulty). The α_2_-TiAl alloys are produced in ingots of 3200 kg [25]. Lapin wrote about extrusion and forging for producing compressor blades for engine testing. Compressor blades were produced by Thyssen, GfE, Leistritz and GKSS for Rolls-Royce using Ti-Al-Nb-(B,C) alloy, but the microstructure was very heterogeneous, and it can be expected that the segregation effects would be even larger for the preparation of bigger components. The boron addition enabled the refinement of the grain size of the as-cast titanium aluminides [19].

The production of Ti-Al-Si alloys by melting metallurgy is very difficult. The disadvantages include the high melting points of intermetallics (titanium silicide Ti_5_Si_3_ melts at the temperature of 2130 °C [12]) and high melt reactivity with the melting crucibles [8], so it is necessary to use Y_2_O_3_ and ZrO_2_ crucibles (which are more expensive than corundum or graphite crucibles). Other problems include the contamination of the melt from the reaction with the atmosphere in the furnace [12]. The Ti-Al-Si alloys have very poor fluidity, so the alloy has many casting defects after casting, for example, pores and cracks in the structure [26,27].

Hard and brittle sharp-edged titanium silicide (Ti_5_Si_3_) phases are formed by melting and casting of Ti-Al-Si alloy. These phases have a negative impact on mechanical properties, especially on the fracture toughness [28]. Hence, there is an effort to eliminate these big brittle phases of titanium silicides. In the previous study, the formation of in situ composites composed of elongated particles of Ti_5_Si_3_ phases in a tough matrix made of TiAl or Ti_3_Al aluminides has been investigated [29]. In the structure, the cracks perpendicular to the direction of solidification were found because of the different coefficient of thermal expansion of Ti_5_Si_3_ in different crystal directions. Therefore, tensile stresses and crack initiation occur [12]. These problems limit the possibility of processing of Ti-Al-Si materials by directional crystallisation [30]. 

Applicability of forming of Ti-Al-Si alloys is also limited due to the low fracture toughness and ductility of the material, which persists even at temperatures above 1000 °C [12]. However, the preparation of Ti-Al alloys with the addition of Si by ingot metallurgy is limited to eutectic and hypoeutectic alloys based on α_2_-Ti_3_Al, since hypereutectic alloys are extremely brittle because of the coarse primary silicides formed upon solidification [10].

In this work, Ti-Al-Si alloys were processed by melting metallurgy in an arc melting furnace. The results were compared with the same alloys prepared by powder metallurgy by the means of a combination of mechanical alloying and spark plasma sintering.

## 2. Materials and Methods 

The Ti-Al-Si alloys (TiAl10Si20, TiAl10Si30, TiAl15Si15, TiAl20Si20 (wt. %)) were prepared by melting metallurgy in the arc melting furnace Bühler MAM-5 in the Institute of Physics of the Czech Academy of Sciences. These compositions have been chosen according to our previous research [26]. The pieces of individual elements (purity 99.95%) were four times re-melted under the argon atmosphere. 

The reference samples were prepared by powder metallurgy. The optimal preparation route was chosen as a combination of mechanical alloying and spark plasma sintering (MA + SPS). The mixture of pure titanium (with a purity of 99.5% and a particle size of 44 µm, STREM CHEMICALS, Newburyport, MA, USA), aluminium (99.62%, 44 µm, STREM CHEMICALS, Newburyport, MA, USA) and silicon (99.5%, 44 μm, Alfa Aesar, Haverhill, MA, USA) powders were filled in a steel vial with steel milling balls (ball-to-powder weight ratio was 60:1) and milled under the argon atmosphere. For the mechanical alloying, a planetary ball mill (PM 100 CM, Retsch, Haan, Germany) was applied. Milling was performed for four hours with rotation velocity of 400 min^−1^. Mechanically alloyed powders were consolidated by spark plasma sintering (FCT Systeme GmbH, Rauenstein, Germany) under the pressure of 80 MPa and the temperature of 1100 °C for 15 min. The applied heating rate and cooling rates were 100 and 50 °C/min, respectively [31]. 

The phase composition was analyzed by the means of the X-ray diffraction analysis (XRD) using a X’Pert Pro (PANalytical, Almelo, Netherlands) X-ray diffractometer with CuKα radiation and a LynxEye XE detector (PANalytical, Almelo, The Netherlands). XRD patterns were evaluated qualitatively by X’Pert HighScore 3.0 software package (PANalytical, Almelo, Netherlands), exploiting PDF-2 2018 database. For the microstructure inspections, samples were ground by P80 to P4000 grinding papers (Hermes Schleifmittel GmbH, Hamburg, Germany) and polished by diamond paste with the particle size of 1–2 μm. The polished samples were etched by Kroll’s reagent (it was prepared in our laboratory) (10 mL HF, 5 mL HNO_3_ and 50 mL H_2_O). The microstructure was investigated by the inverted optical microscope Olympus PME3 (Olympus, Prague, Czech Republic) and documented by the Carl Zeiss AxioCam ICc3 (Carl Zeiss, Jena, Germany) digital camera and AxioVision software package (version 4.8.2, Carl Zeiss, Jena, Germany). Porosity was evaluated by Lucia 4.8 image analyser (Laboratory Imaging, Prague, Czech Republic). TESCAN VEGA 3 LMU (TESCAN, Brno, Czech Republic) electron microscope with EDS analyser Oxford Instruments X-max 20 mm^2^ (Oxford Instruments, High Wycombe, UK) (SEM-EDS) was used for the deeper microstructure investigation and the identification of present phases on the micrographs. 

Compressive strength tests were performed with the use of the universal testing machine LabTest 5.250SP1-VM (LaborTech, Opava, Czech Republic). Values of ultimate compressive strength were determined from the measured stress-strain curves. Vickers hardness with a load of 5 kg (HV 5), 100 grams (HV 0.1) and 50 grams (HV 0.05) was measured on the polished samples (10 measurements on each specimen). Fracture toughness was measured by Vickers indentation method with the load of 1 kg on microhardness tester Future-Tech FM-700 (Future-Tech, Kawasaki, Japan). Indentions were observed by the means of the above-mentioned Olympus PME3 microscope. Fracture toughness was calculated using Palmqvist’s Equation (1):

(1) Kc=0.016·(EHV)12·(Fc32)

where E is modulus of elasticity (GPa), HV is Vickers hardness (GPa), F is load (N), and c is half of the crack length after indention (mm).

## 3. Results

**Figure 1:**
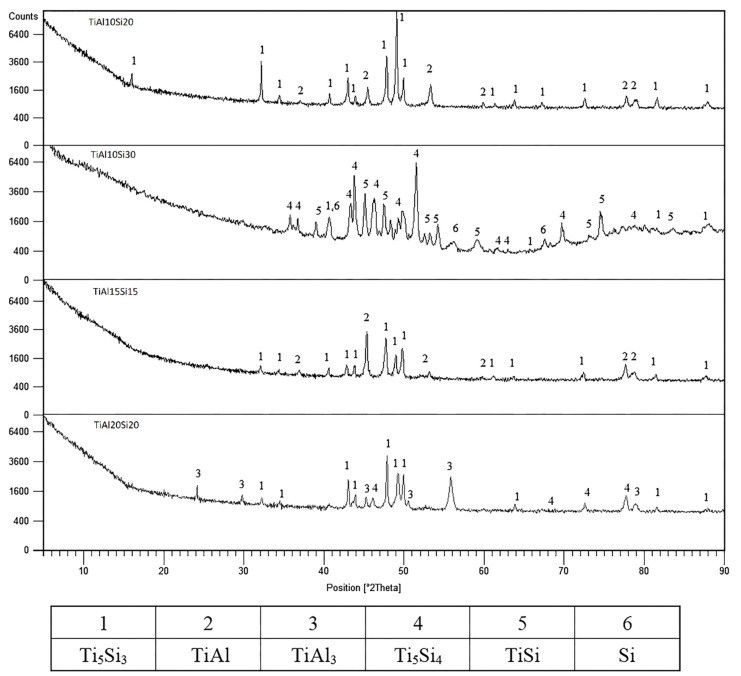
XRD patterns of Ti-Al-Si alloys processed by melting metallurgy.

The phase composition of as-cast Ti-Al-Si alloys is displayed in Figure 1. The TiAl10Si20 and TiAl15Si15 alloys consists of titanium silicide (Ti_5_Si_3_) and titanium aluminide (TiAl). Titanium silicide TiSi, Ti_5_Si_4_ and pure silicon were, as expected, found in the TiAl10Si30 alloy due to the higher amount of silicon in the alloy. The TiAl20Si20 alloy is composed of Ti_5_Si_3_ and Ti_5_Si_4_ silicides in TiAl_3_ aluminide matrix.

**Figure 2:**
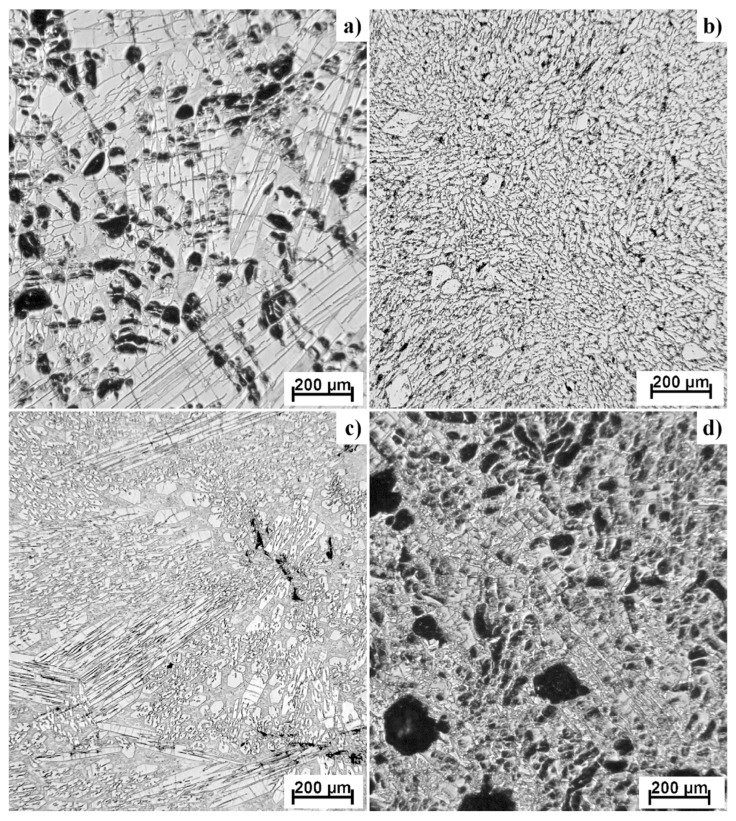
Microstructures of Ti-Al-Si alloys processed by melting metallurgy (light microscopy): (**a**) TiAl10Si20, (**b**) TiAl10Si30, (**c**) TiAl15Si15, (**d**) TiAl20Si20 (black areas are pores, lighter grey particles are titanium silicides, darker grey ones are titanium aluminides).

Figure 2 shows the microstructure of Ti-Al-Si alloys prepared by arc melting. The as-cast alloys have coarse dendritic structures. As-cast TiAl10Si20 alloy is characterized by a very porous structure, as well as TiAl20Si20. The pores (black spots on the photos) are concentrated largely in the center of the sample and they are irregular in shape. Alloys TiAl10Si30 and TiAl15Si15 have much smaller pores. The particles of titanium silicides are very coarse in each alloy, and they have various local characters of morphology. The microstructure is characterized by primary silicides, silicides with fibrous and lamellar morphology and fine eutectic structure. The colonies of titanium silicides are differently oriented, depending on the local direction of heat transfer, which causes the elongation of silicides.

**Figure 3:**
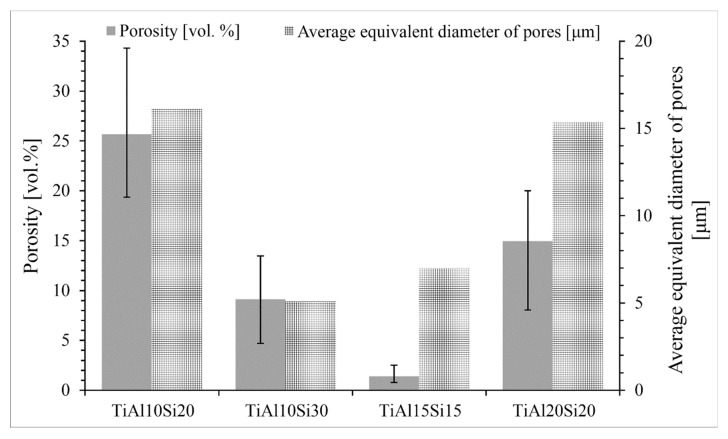
Porosity vol. and average equivalent diameter of pores of Ti-Al-Si alloys processed by melting metallurgy. no subscript

Figure 3 shows the porosity and average equivalent diameter of pores of Ti-Al-Si alloys prepared by melting metallurgy. The big differences between the values of porosity are given by preparation techniques. During the fast cooling, the gasses are closed in the middle of the sample and formed the middle porosity. At the edges of sample, the porosity is minimal.

**Figure 4:**
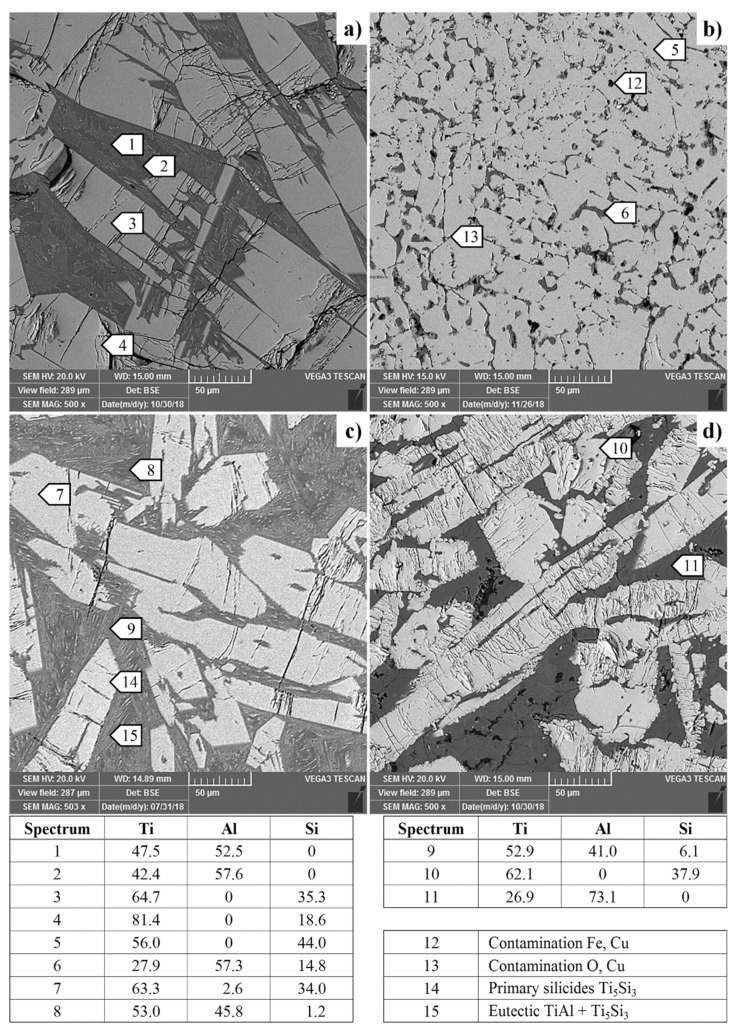
Microstructure of Ti-Al-Si alloys processed by melting metallurgy (light microscopy): (**a**) TiAl10Si20, (**b**) TiAl10Si30, (**c**) TiAl15Si15, (**d**) TiAl20Si20 (lighter particles are titanium silicides, darker ones are titanium aluminides) (composition in atomic %).

**Figure 5:**
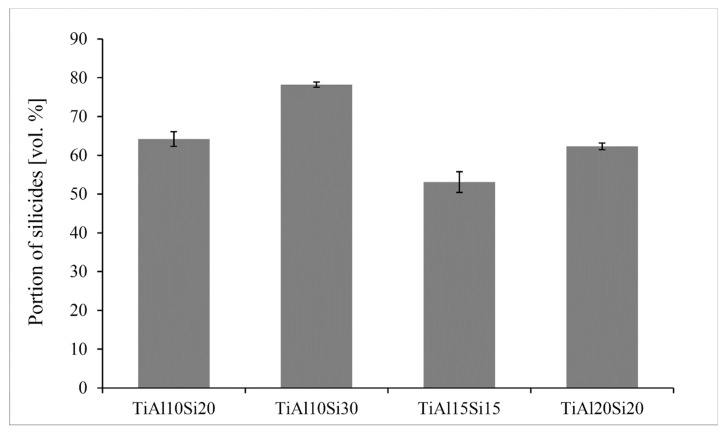
Amount of silicides in Ti-Al-Si alloys processed by melting metallurgy.

Figure 4 presents the SEM micrographs of Ti-Al-Si alloys acquired in a backscattered electrons (BSE) regime. Except for the TiAl10Si30, all alloys have multiple cracks in the titanium silicides. Composition of each phase (TiAl, Ti_5_Si_3_) in TiAl10Si20 alloy corresponds well with the phase diagram. Only spectrum four contains a local lower amount of silicon, which was detected near the crack. TiAl10Si30 alloy has the finest particles of titanium silicides. At the same time, TiAl10Si30 alloy has the lowest amount of aluminide matrix (Figure 5). It is due to the highest amount of silicon in the basic chemical composition and the higher ratio between aluminium and silicon in the alloy. The TiAl10Si30 alloy processed by casting exhibited the presence of areas of aluminides, containing higher degree of substitution of aluminium by silicon (Figure 4b). In addition, TiAl10Si30 alloy are contaminated by iron, copper and oxygen from the preparation route. In TiAl15Si15 alloy, the chemical composition of TiAl and Ti_5_Si_3_ phase corresponds well with the phase diagram, i.e., the phases are minimally substituted by other elements. According to the EDS (Energy Dispersive Spectroscopy) analysis (Spectrum 11) and Ti-Al phase diagram, TiAl20Si20 should contain aluminide phase (TiAl_3_), which was confirmed by XRD diffraction. 

**Figure 6:**
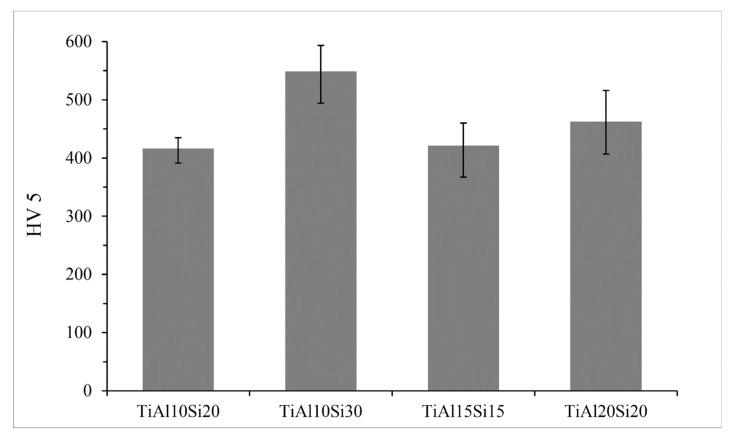
Hardness of Ti-Al-Si processed by melting metallurgy.

The hardness of the Ti-Al-Si alloys (Figure 6) prepared by melting metallurgy varies between 416 and 549 HV 5. TiAl10Si30 alloy with the highest amount of silicon has a higher amount of hard silicides (Figure 5) and, hence, it reaches the highest values of hardness. 

**Figure 7:**
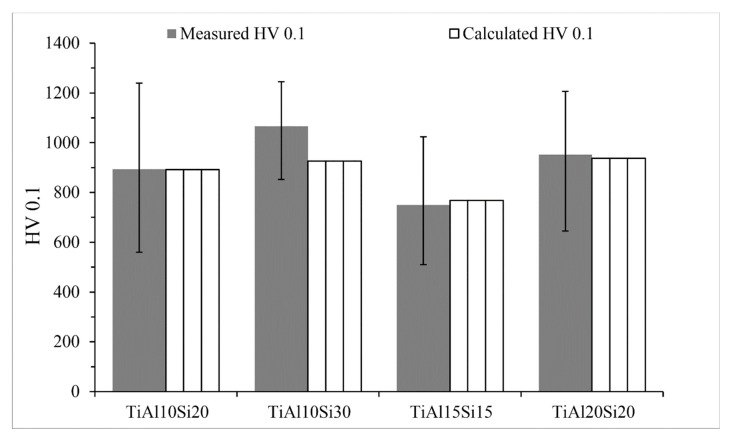
Microhardness of the Ti-Al-Si alloys processed by melting metallurgy.

The microhardness of the Ti-Al-Si alloys (Figure 7) is in the range between 750 and 1066 HV 0.1 and TiAl10Si30 alloy achieves the highest hardness. It is caused by the fact that silicon in alloy is bonded in silicides (Ti_5_Si_3_, Ti_5_Si_4_ and TiSi), which are harder than the contained aluminides. The predicted hardness was calculated by Equation (2). The calculated hardness values of Ti-Al-Si alloys are compared with the measured ones in Figure 7. The values are comparable, only TiAl10Si30 alloy has lower values of calculated hardness than hardness, which was measured.

(2) $$w_{\left(Ti-Si\right)}\cdot HV_{\left(Ti-Si\right)}+w_{\left(Ti-Al\right)}\cdot HV_{\left(Ti-Al\right)}=HV_{\left(Ti-Al-Si\right)}$$

where w_(Ti-Si)_ is an area fraction of titanium silicides, HV_(Ti-Si)_ is the Vickers hardness of titanium silicides, w_(Ti-Al)_ is an area fraction of titanium aluminides, HV_(Ti-Al)_ is Vickers hardness of titanium aluminides, and HV_(Ti-Al-Si)_ is the hardness of the product.

**Figure 8:**
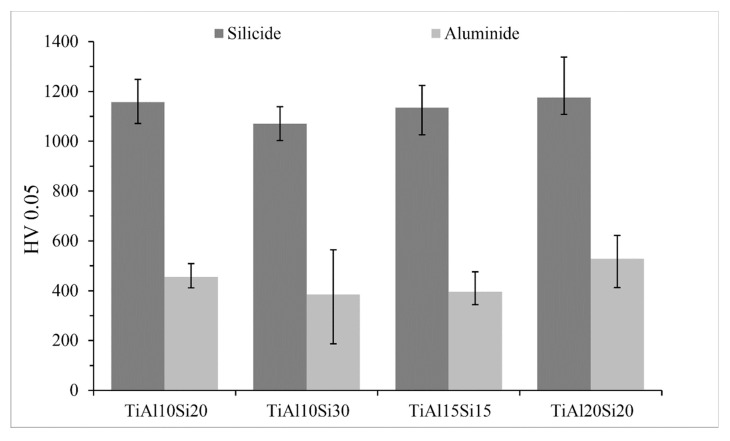
Comparison of microhardness of silicide and aluminide phase.

Figure 8 shows the comparison of microhardness of silicide and aluminide phase. Silicide phase hardness varies between 1070 and 1175 HV 0.05, the hardness of the aluminide phase varies between 200 and 530 HV 0.05. The big variations in the hardness of the aluminide phase are given by the substitution of silicon in titanium aluminide and the formation of small particles of titanium silicides in Ti-Al matrix. Titanium aluminide phase TiAl_3_ present in TiAl20Si20 alloy has a higher hardness than TiAl phase. Values of the hardness of titanium silicides show only minor variations (Figure 8), so it is possible to say that silicides Ti_5_Si_3_ and Ti_5_Si_4_ have the same hardness, but titanium silicide TiSi present in TiAl10Si30 alloy has a slightly lower hardness.

**Figure 9:**
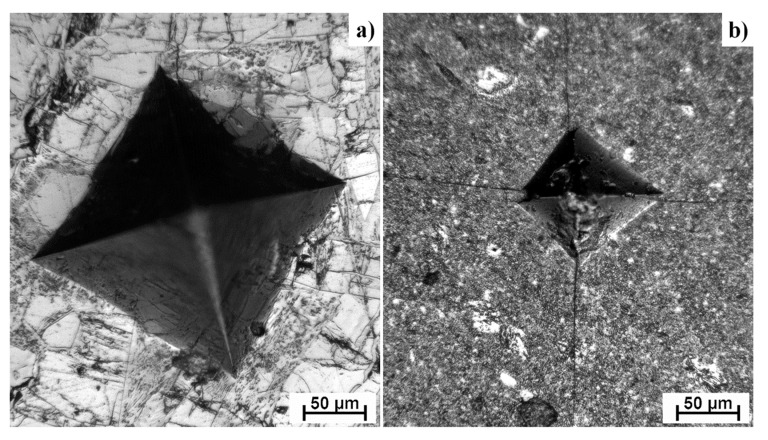
Cracks after indentation for calculating the fracture toughness of TiAl15Si15 alloy processed by: (**a**) melting metallurgy, (**b**) powder metallurgy.

**Figure 10:**
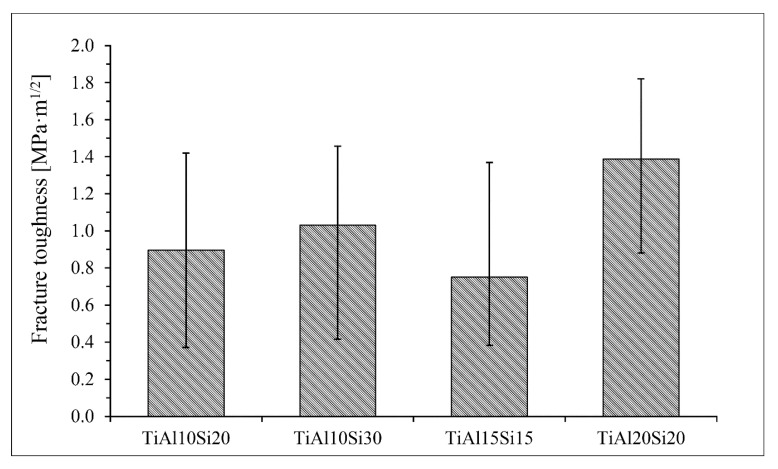
Fracture toughness of silicide phases in Ti-Al-Si alloys processed by melting metallurgy.

**Figure 11:**
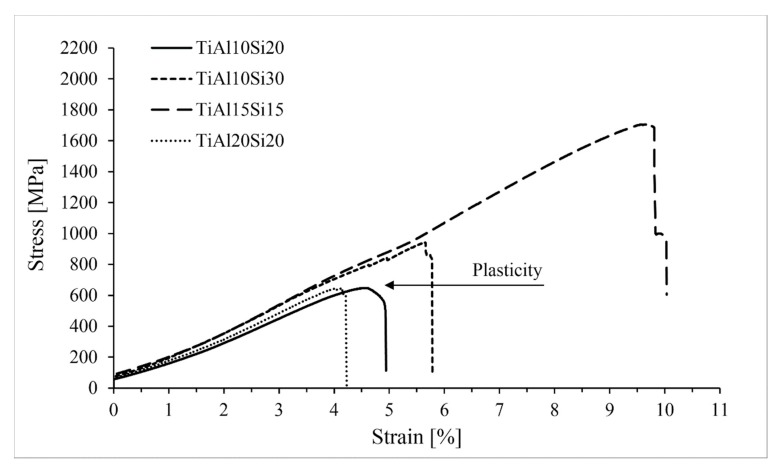
Compression tests of Ti-Al-Si alloys processed by melting metallurgy.

Intermetallics belong to the materials commonly referred as brittle. For these materials classes, the fracture toughness and compressive strength are the measures for potential use in the practice. Fracture toughness of Ti-Al-Si alloys processed by melting metallurgy was measured from the ten indentions into the silicide phase of each sample using Palmqvist’s equation. Since titanium silicides are brittle, the cracks were formed during the indention. All of the alloys possess lower fracture toughness than the technical ceramics, such as alumina or silicon carbide having fracture toughness of around 4 MPa m^1/2^ [32]. Crack propagation was stopped by the present aluminide phase at the TiAl15Si15 alloy prepared by arc melting. On the other hand, the crack spreads through the sample in case of the same alloy prepared by powder metallurgy (Figure 9). Therefore, the fracture toughness of the titanium silicides was measured (Figure 10). It is possible to say that a coarser structure is conducive to an increase on the toughness of the material and the samples are less brittle. The signs of the plasticity of the as-cast alloys are also shown in the deformation curves in compression (Figure 11). The TiAl15Si15 alloy achieves the higher ultimate tensile strength in compression—1700 MPa. This value of UTS is comparable with the same alloy processed by the combination of mechanical alloying and spark plasma sintering [31].

**Figure 12:**
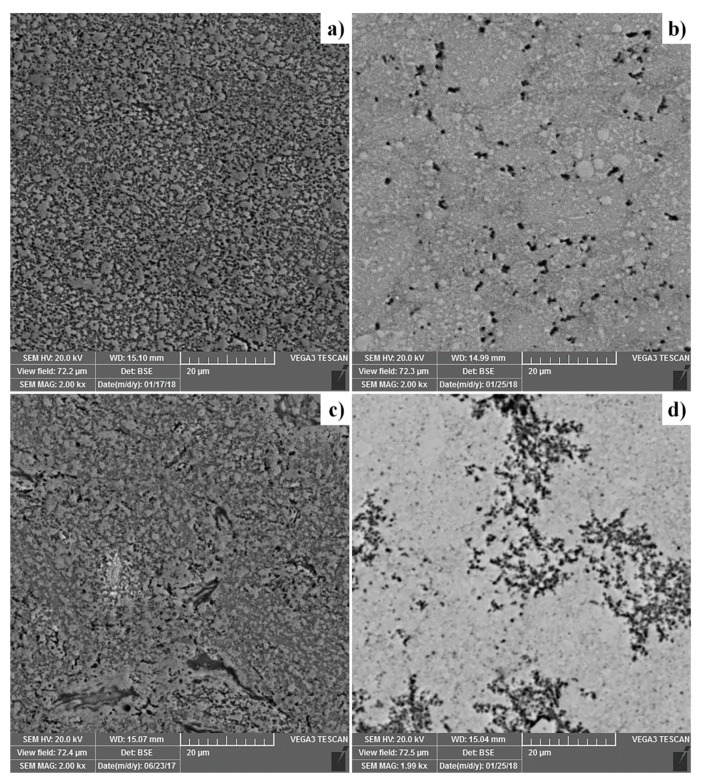
Microstructure of Ti-Al-Si alloys processed by powder metallurgy using mechanical alloying and Spark Plasma Sintering (scanning electron microscopy): (**a**) TiAl10Si20, (**b**) TiAl10Si30, (**c**) TiAl15Si15, (**d**) TiAl20Si20.

The reference samples of Ti-Al-Si alloys were prepared by powder metallurgy, by mechanical alloying and Spark Plasma Sintering (MA + SPS). SEM micrographs of the MA + SPS alloys are presented in Figure 12. The TiAl10Si20 alloy is characterized by a very homogeneous well-refined structure, and also the most fine-grained, with the Ti_5_Si_3_ strengthening phase in TiAl matrix. TiAl10Si30 is composed by Ti_5_Si_3_ and Ti_5_Si_4_ silicides in TiAl and TiAl_2_ matrix. The TiAl15Si15 alloy contains a small amount of Ti_2_Al phase. TiAl20Si20 alloy is composed of Ti_5_Si_3_, Ti_5_Si_4_, and TiSi_2_ silicides, while the higher ratio between aluminium and titanium unbound in silicides leads to the single-phase TiAl_3_ matrix. The sample also contains iron contamination originating from the milling steel vial.

**Figure 13:**
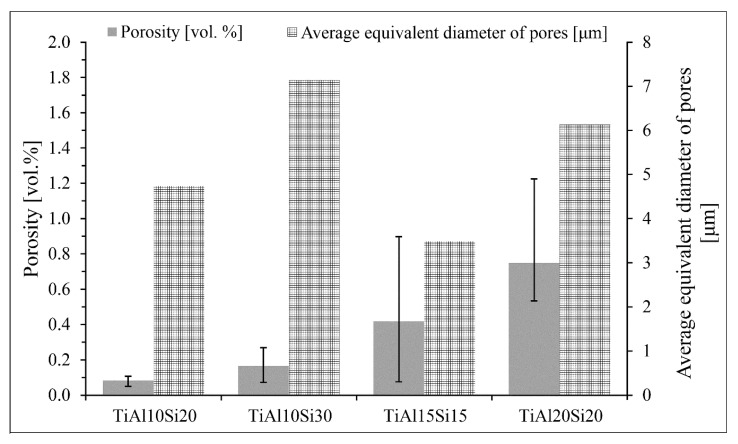
Porosity vol. and average equivalent diameter of pores of Ti-Al-Si alloys processed by powder metallurgy.

Alloys processed by mechanical alloying and the spark plasma sintering method are characterized by lower porosity (Figure 13) than the alloys prepared by arc melting (Figure 3). The porosity of Ti-Al-Si alloys processed by powder metallurgy is lower than 1 vol. %. The pores are uniformly distributed (Figure 12).

**Figure 14:**
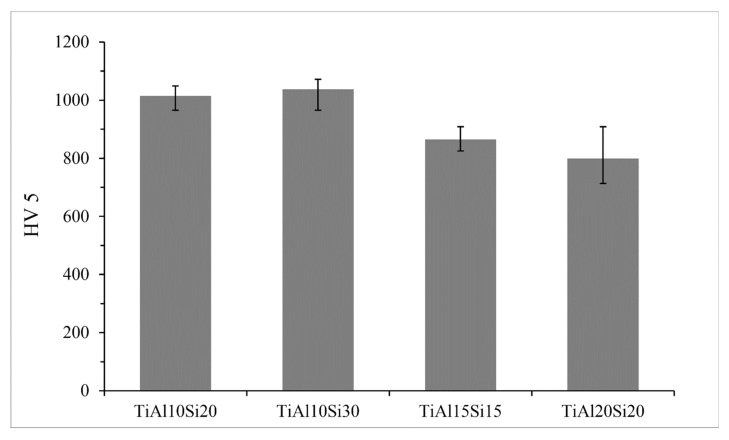
Hardness of Ti-Al-Si processed by powder metallurgy.

The hardness of the Ti-Al-Si alloys (Figure 14) prepared by powder metallurgy varies between 800 and 1037 HV 5. These values of hardness are higher than values of Ti-Al-Si alloys processed by arc melting. The higher hardness corresponds to more homogeneous and fine-grained crackless microstructure. TiAl10Si30 and TiAl10Si20 reach the highest values of hardness.

**Figure 15:**
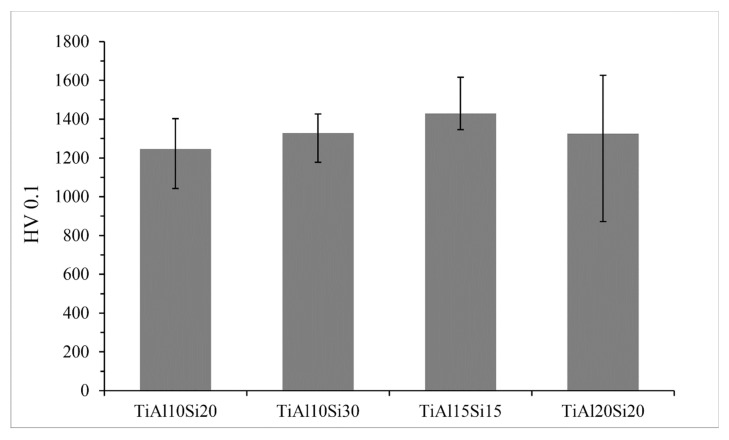
Microhardness HV 0.1 of Ti-Al-Si processed by powder metallurgy (mechanical alloying and Spark Plasma Sintering).

The microhardness of the Ti-Al-Si alloys prepared by powder metallurgy (Figure 15) varies between 1246 and 1430 HV 0.1. The measured microhardness is higher than the microhardness of Ti-Al-Si materials processed by melting metallurgy.

**Figure 16:**
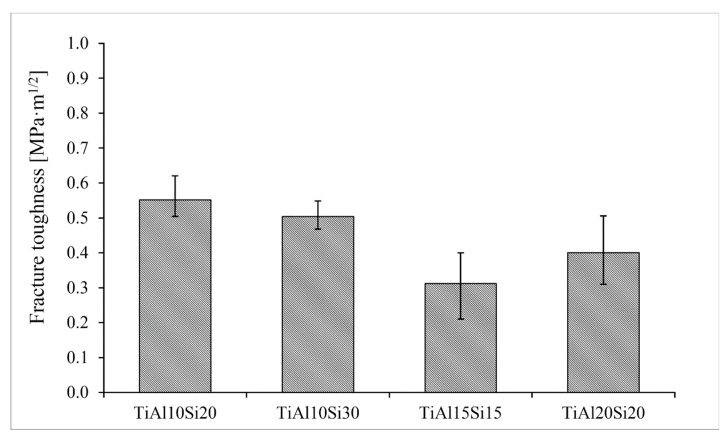
Fracture toughness of silicide phases in Ti-Al-Si alloys processed by melting metallurgy.

The fracture toughness of Ti-Al-Si alloys prepared by powder metallurgy was measured by the indentation and was calculated by Palmqvist’s Equation. All of the alloys have low values of fracture toughness, because the intermetallic phases present in these alloys are very brittle (Figure 16). The alloys processed by powder metallurgy have lower values of fracture toughness than the same alloys prepared by melting metallurgy.

**Figure 17:**
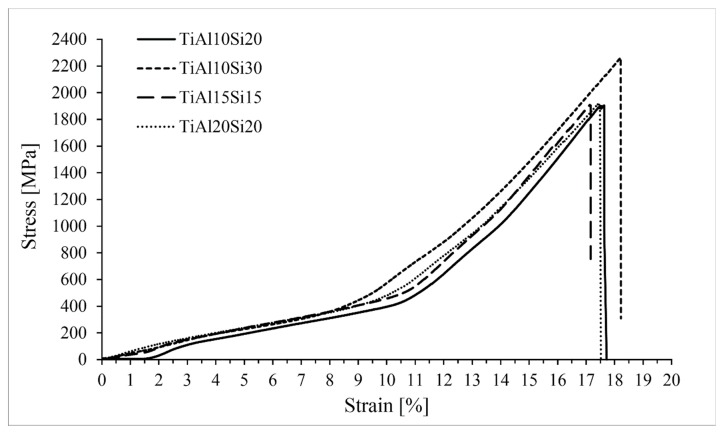
Compression tests of Ti-Al-Si alloys processed by melting metallurgy.

The Ti-Al-Si alloys processed by powder metallurgy achieve higher ultimate tensile strength in comparison with the same alloys processed by melting metallurgy (Figure 17). The alloys have better values due to the more homogeneous and fine-grained structure. 

## 4. Discussion

Table 1 shows the comparison of the phase composition of the Ti-Al-Si materials processed by melting and powder metallurgy. The phase composition of TiAl10Si20 alloy processed by both the arc melting and the MA + SPS is in good agreement with the equilibrium phase diagram of Ti-Al-Si ternary system [31,33]. The same results were obtained by reactive sintering followed by spark plasma sintering (RS + SPS) described in our previous article [26]. Another titanium silicide, TiSi, was found in the TiAl10Si30 alloy due to the higher amount of silicon in the alloy. Formation of TiSi corresponds to the equilibrium phase diagram [33] and this titanium silicide was also found in the TiAl10Si30 alloy prepared by RS + SPS [26]. As-cast TiAl15Si15 alloy has the same phase composition as in the equilibrium phase diagram, but the alloy prepared by MA + SPS contains a small amount of Ti_2_Al phase. The TiAl20Si20 alloy contains a combination of silicides Ti_5_Si_3_, Ti_5_Si_4_, and TiSi_2_ (by type of preparation) and a single-phase TiAl_3_ matrix caused by the higher ratio between aluminium and titanium unbound in silicides.

Mechanical alloying causes that the aluminium in titanium aluminide and silicon in titanium silicides are strongly substituted by silicon and aluminium, respectively. Authors described that Ti(Al,Si) supersaturated solid solutions are formed after at least 10 or 20 h of milling [34,35], but it is shown in this work that intermetallic phases are formed at the shorter time of mechanical alloying (after 4 h).

The Ti-Al-Si alloys prepared by melting metallurgy (Figure 2) have a very coarse structure with pores, which are concentrated in the centre of the sample. The coarse particles of titanium silicides are oriented depending on the local direction of heat transfer, which causes the elongation of silicides. These silicides are cracked due to the high cooling rate after melting [36]. The central porosity is given by the processing technology. Gases are trapped in the sample as a result of evaporation of the elements (mostly aluminium) during the exothermic formation of intermetallics and relatively rapid cooling. The highest cooling rate is on the surface of the samples, so the samples solidified from the surface to the core. The gases are trapped in the middle sample and they cannot escape from the sample because of the fast solidification of the surface. Therefore, the porosity of the samples is concentrated in the centre of the samples.

Powder metallurgy (MA + SPS) results in a very fine microstructure of the investigated alloys (Figure 12). Authors described that mechanical alloying is a very effective process in obtaining of nanosized grains [37,38]. Refining of Ti_5_Si_3_ crystallites is probably due to a strong deformation and consequent recrystallization. The distribution of titanium silicides is much more homogeneous because the brittle powder is crushed to very fine particles during the first step of mechanical alloying. The elements in the obtained compounds are substituted mutually, exceeding the equilibrium solubility limits [39]. Figure 8 compares the hardness of silicide and aluminide phase in as-cast Ti-Al-Si alloys. Titanium aluminide TiAl_3_ is harder than TiAl. This confirms the fact than TiAl_3_ has covalent bond and the TiAl phase has a metallic bond, which causes the higher plasticity [40,41]. The hardness of titanium aluminide TiAl_3_ is referred to as 450 HV [42], TiAl phase around 300 and 350 HV 5 [43]. The hardness of Ti_5_Si_3_ is referred to as 970 HV [44]. Calculated and measured results in Ti-Al-Si alloys are a little bit higher than the values reported in the literature. Hardness values of titanium silicides have a very small scattering of values, so it is possible to say that they have isotropic mechanical properties [36].

Powder metallurgy (MA + SPS) resulted in an enormous porosity reduction, which decreased from 26 vol. % in the case of as-cast TiAl10Si20 to under 1 vol. % by the same alloy (Figure 3, Figure 13). The other alloys recorded a similar decline. Also, the average equivalent diameter of pores decreases from the tens of micrometres to micrometre units. The applied the load, the sintering temperature and duration are the most important parameters, which influence the microstructure, porosity and pore size of the resulting compact sample. The better compaction of the powder and the smaller porosity and pore size are obtained from the higher temperature and applied load, but it may cause the grain coarsening [45]. In previous works, the optimal conditions of spark plasma sintering were studied so that the porosity and pore size were as low as possible [46].

The hardness, microhardness, and ultimate tensile strength (UTS) of the Ti-Al-Si alloys prepared by powder metallurgy (MA + SPS) are higher than the same alloys prepared by arc melting. The higher hardness and UTS corresponds to a more homogeneous and crackless microstructure, which is described above. The force during the mechanical alloying deforms the powder particles plastically, which leads to work hardening and fracture. Hardening, in combination with a fine-grained structure, increases the material hardness [45], but lowers the plasticity.

## 5. Conclusions

In this work, Ti-Al-Si were prepared by arc melting and powder metallurgy. Both technologies were compared. Ti-Al-Si alloys processed by arc melting are characterized by a porous structure with very coarse titanium silicide particles in a titanium aluminide matrix. Titanium silicides are full of cracks due to the relatively fast cooling. Reference samples processed by mechanical alloying and spark plasma sintering are characterized by a more homogeneous and finer structure with low porosity. These properties guarantee excellent mechanical properties. Vickers hardness of MA+SPS samples reaches double values, but Ti-Al-Si alloys processed by powder metallurgy are very brittle. The fracture toughness is worse than for technical ceramics. On the other hand, arc-melted samples have immeasurable fracture toughness using Palmqvist’s method, the Vickers indentions do not cause the cracks, and crack propagation is stopped by the aluminide phase. For this reason, it is reasonable to say that TiAl15Si15 alloy prepared by melting metallurgy is the most promising alloy, as the formation of a coarse-grained structure improves the fracture toughness, the alloy is more ductile, and comparable ultimate tensile strength (as MA+SPS) predetermines this alloy for use in applications where high hardness is not the most important. However, it is necessary to improve the melting process (to optimize the conditions of melting and casting), or to use the hot isostatic pressing of the castings, in order to decrease the porosity of the material.

## Figures and Tables

**Table 1:** Comparison of phase composition of Ti-Al-Si alloys (MA = mechanical alloying, RS = reactive sintering, SPS = Spark Plasma Sintering).

Alloy	Phase Diagram [27]	Melting	MA + SPS	RS + SPS [21]
TiAl10Si20	Ti_5_Si_3_, TiAl	Ti_5_Si_3_, TiAl	Ti_5_Si_3_, TiAl	Ti_5_Si_3_, TiAl
TiAl10Si30	TiSi, Ti_5_Si_4_, TiAl_3_	TiSi, Ti_5_Si_3_, Ti_5_Si_4_, Si	Ti_5_Si_3_, TiAl, TiAl_2_, Ti_5_Si_4_	TiSi, Ti_5_Si_3_, TiAl_3_, Ti_2_Al, Si
TiAl15Si15	Ti_5_Si_3_, TiAl	Ti_5_Si_3_, TiAl	Ti_5_Si_3_, TiAl, TiAl_2_	Ti_5_Si_3_, TiAl
TiAl20Si20	Ti_5_Si_3_, TiAl_3_	Ti_5_Si_3_, TiAl_3_, Ti_5_Si_4_	Ti_5_Si_3_, TiAl_3_, Ti_5_Si_4_, TiSi_2_	Ti_5_Si_3_, TiAl_3_, Ti_5_Si_4_, TiSi_2_
